# Cell-Based Therapies for Tissue Fibrosis

**DOI:** 10.3389/fphar.2017.00633

**Published:** 2017-09-22

**Authors:** Rebecca Lim, Sharon D. Ricardo, William Sievert

**Affiliations:** ^1^The Ritchie Centre, Hudson Institute of Medical Research, Clayton VIC, Australia; ^2^Department of Obstetrics and Gynaecology, Monash University, Melbourne VIC, Australia; ^3^Monash Biomedicine Discovery Institute, Monash University, Melbourne VIC, Australia; ^4^Centre for Inflammatory Diseases, School of Clinical Sciences, Monash University, Melbourne VIC, Australia; ^5^Gastroenterology and Hepatology Unit, Monash Health, Melbourne VIC, Australia

**Keywords:** fibrosis, stem cells and regenerative medicine, cell therapy, mesenchymal stem cells, progenitor cells

## Abstract

The development of tissue fibrosis in the context of a wound-healing response to injury is common to many chronic diseases. Unregulated or persistent fibrogenesis may lead to structural and functional changes in organs that increase the risk of significant morbidity and mortality. We will explore the natural history, epidemiology, and pathogenesis of fibrotic disease affecting the lungs, kidneys, and liver as dysfunction of these organs is responsible for a substantial proportion of global mortality. For many patients with end-stage disease, organ transplantation is the only effective therapy to prolong life. However, not all patients are candidates for the major surgical interventions and life-long immunosuppression required for a successful outcome and donor organs may not be available to meet the clinical need. We will provide an overview of the latest treatment strategies for these conditions and will focus on stem or progenitor cell-based therapies for which there is substantial pre-clinical evidence based on animal models as well as early phase clinical trials of cell-based therapy in man.

## Introduction

An appropriate response to injury is required for homeostasis. While injury may take many forms, the repair response is typically generic. An understanding of aberrant wound repair has direct relevance to human disease given that organ fibrosis has been estimated to contribute to 45% of all-cause human mortality (Wynn, 2004). While large, this statistic should not be surprising given the significance of fibrosis in chronic diseases affecting multiple organs (**Table 1**). Despite an extensive understanding of fibrogenesis in response to injury, no effective anti-fibrotic therapies are currently available. The highly conserved wound healing response is also highly redundant with multiple overlapping pathways suggesting that inhibition of a single candidate molecule or pathway is insufficient and new approaches are required. Based on this notion, cell-based therapies with the potential to alter multiple therapeutic targets are gaining popularity. A broad discussion of all stem cell types is beyond the focus of this mini-review. We will concentrate on mesenchymal stem cells (MSCs), which form the largest experience in cell therapy, as well as our work with placental stem cells.

**Table 1:** Fibrosis as a major component of chronic diseases.

Organ	Conditions
Skin	Systemic sclerosis (may involve lung and kidney)
	Keloids, burns
Lung	Idiopathic pulmonary fibrosis
	Interstitial lung disease (multiple aetiologies)
	Cystic fibrosis (may involve pancreas)
Heart, blood vessels	Congestive heart failure/cardiac fibrosis
	Atherosclerosis (affects multiple organs)
Liver	Cirrhosis (multiple aetiologies)
	Hepatorenal fibrocystic diseases
Intestine	Crohn’s disease
	Post-operative adhesions
Pancreas	Chronic pancreatitis
Kidney	End-stage renal disease (diabetes or hypertension)
	Renal interstitial fibrosis
Immune system	Chronic graft vs. host disease
Musculo-skeletal system	Rheumatoid arthritis (may involve lung)
	Ankylosing spondylitis
	IgG4-related retroperitoneal fibrosis

## Lung Fibrosis

### Epidemiology, Burden of Disease, and Natural History

Pulmonary fibrosis is a family of over 200 chronic lung diseases stemming from multiple underlying causes including autoimmune diseases such as scleroderma and rheumatoid arthritis. Pulmonary fibrosis may be a consequence of environmental exposure to inhaled dust, bacteria, or molds, but can also arise following exposure to cancer treatments such as radiation therapy or chemotherapy using bleomycin or methotrexate. However, idiopathic pulmonary fibrosis (IPF), a type of pulmonary fibrosis where the cause is unknown occurs in 3–9 per 100,000 people annually based on conservative estimates from Europe and North America (Hutchinson et al., 2015). The incidence of IPF is increasing globally, comparable to many cancers (Hutchinson et al., 2015). A low incidence of IPF in some countries may reflect exclusion of milder cases or inconsistent classification. The severity of reported disease appears to be greater in East Asia, where the majority of cases were recorded as “unspecified interstitial lung disease” rather than IPF (Munakata et al., 1994; Ohno et al., 2008; Lai et al., 2012; Han et al., 2013).

### Current Clinical Management

The clinical progression of IPF is often slow and gradual but an accelerated decline has been reported in some patients, associated with episodes of acute respiratory exacerbations. The median survival rates are historically poor at 2–3 years, with 5-year survival ranging between 30 and 50% (Bjoraker et al., 1998; Mapel et al., 1998; Rudd et al., 2007; Raghu et al., 2011). To date, lung transplantation remains the only intervention with proven benefit. Corticosteroid use is discouraged due to the association between steroid use and survival rates following acute exacerbations (Papiris et al., 2015).

While drugs such as nintedanib and pirfenidone appear to reduce disease progression, widespread usage is unlikely due to their high cost and conflicting data surrounding clinical efficacy. Currently, the proposed use of pirfenidone is to bridge between diagnosis and lung transplantation (Delanote et al., 2016). Nintedanib has also been found to prevent disease progression, and both drugs are comparable in terms of their estimated costs and health-related quality of life benefits (Rinciog et al., 2017). However, neither is curative and their cost is high (£100,000 per QALY). Thus, there is a need to identify alternative therapies.

### Pathophysiology

Historically, IPF was believed to be an inflammatory disorder that progresses to fibrosis. The failure of anti-inflammatory and immunosuppressive therapeutic strategies triggered the need for reassessment (Selman et al., 2001; Raghu et al., 2012). The current consensus is that IPF is a consequence of multiple interacting genetic and environmental risk factors, with repeated damage and premature aging of alveolar epithelial cells (AECs) in genetically susceptible individuals (Wells and Maher, 2017). One robust genetic linkages to IPF is *MUC5B* polymorphism; however, the role of this gene in IPF pathogenesis remains undefined (Conti et al., 2016; Nakano et al., 2016). Unsurprisingly, the prototypic pro-fibrotic transforming growth factor-β (TGFβ) plays a central role in IPF, and while its function is well described, the source of excess TGFβ and activation of its latent form are poorly understood. A recent study by Froese et al. (2016) uncovered a role for mechanotransduction in TGFβ activation, unique to fibrotic lungs, suggesting that the physical stiffness of IPF lungs and mechanical forces applied to fibrotic lungs may contribute to disease perpetuation. Premature aging, telomere shortening, and alveolar senescence are also thought to contribute to IPF pathogenesis. Telomere dysfunction in AECs but not collagen-producing cells is responsible for age-related lung fibrosis (Naikawadi et al., 2016). When telomere dysfunction was conditionally induced in type 2 AECs (AEC2) in mice, an AEC2-induced cytokine response was detected and when challenged with bleomycin, a 100% mortality rate was observed, supporting the critical role of telomere function in AEC2 for alveolar repair (Alder et al., 2015). Given the role of AEC2 as alveolar progenitor cells, Adler et al. concluded that alveolar stem cell failure might contribute to lung fibrosis. These observations have led some to postulate that a regenerative approach is required (Chambers and Hopkins, 2013).

### Cell Therapies for IPF

To date there are six Phase I/II clinical trials (ClinicalTrials.gov) using stem cells for IPF, predominantly allogeneic bone marrow-derived MSCs (NCT01919827, NCT02594839, and NCT02013700). However, placenta and adipose tissue-derived MSCs have also been tested (NCT01385644 and NCT02135380). Interest in MSC-based therapies is attributed to their reported immunomodulatory and anti-fibrotic properties exerted through paracrine mediators. For example, there is recent evidence that MSC can reduce ER stress, thereby improving survival and function of AEC2 through the release of hepatocyte growth factor (Nita et al., 2017). One current clinical trial is aimed at a specific subset (p63+/Krt5+) of the patient’s own lung stem cells (NCT02745184), with the purpose of encouraging cell engraftment and restoring the lost p63+/Krt5+ distal airway stem cells in the fibrotic lung (Zuo et al., 2015). The outcomes of two trials have been published. Allogeneic placental MSCs given at a dose of 1 or 2 × 10^6^ cells/kg body weight were well tolerated in moderate to severe IPF (Chambers et al., 2014). Similarly, a single infusion of 20, 100, or 200 million allogeneic bone marrow MSCs was well tolerated by patients with mild to moderate IPF (Glassberg et al., 2016). While these safety outcomes are encouraging, clinical efficacy remains to be determined.

## Kidney Fibrosis

### Epidemiology and Pathogenesis of Fibrosis in Kidney Disease

The epidemic of chronic kidney disease (CKD) and end-stage renal failure (ESRF) is a crisis for global healthcare. There is urgent need for new therapeutic options considering the high morbidity of dialysis, extensive healthcare costs, and donor-kidney shortages. Known risk factors for CKD include age, hypertension, obesity, and diabetes (McMahon et al., 2014).

Regardless of etiology, the common end-point of kidney injury is fibrosis leading to CKD development (Samarakoon et al., 2012). An excessive inflammatory and fibrotic response to injury results in decreased renal function as the renal tubules are damaged by scar tissue (Hewitson, 2009). Following initial renal injury, endogenous kidney cells release pro-inflammatory chemokines (Balasubramanian, 2013) that recruit inflammatory cells, activating fibroblasts, and causing tubular dilation (Meran and Steadman, 2011). The recruited immune cells release further inflammatory cytokines including those from the TGFβ superfamily and mitogen-activated protein kinases (MAPK/ERK) that activate fibrotic genes through SMAD signaling (Chevalier et al., 2010), leading to interstitial fibrosis and extracellular matrix accumulation. While inflammation and the TGFβ pathway are essential for normal kidney development and homeostasis, unopposed expression results in a harmful cycle of injury as seen in CKD (Schnaper et al., 2009).

### Potential of Cell-Based Therapies for Kidney Disease

Stem or progenitor cell therapies offer a strategy for modulating CKD progression by suppressing multiple pathogenic pathways and promoting pro-regenerative mechanisms. MSCs are pursued as a therapeutic tool as they are immunomodulatory, easily obtainable from bone marrow, and can be expanded in culture for use in the clinic (Yagi et al., 2010). MSCs elicit endogenous repair through paracrine and/or endocrine mechanisms that modulate the immune response, ultimately allowing for cellular replacement. In pre-clinical studies we have demonstrated that MSCs have immunomodulatory properties, and secrete anti-inflammatory cytokines that promote inhibition of pro-inflammatory cytokines (Wise et al., 2014; Huuskes et al., 2015; Wise et al., 2016). MSCs have been used in experimental and clinical settings to improve diabetes and diabetic complications including kidney fibrosis. Recent clinical trials show that MSCs are safe and well tolerated in diabetes (Skyler et al., 2015); however, the diabetic microenvironment and/or comorbidities alter the quality or efficacy of MSCs following transplantation. Further mechanistic studies are needed to understand how MSCs protect against fibrotic injury and to improve efficacy following cell transplantation to overcome the transient clinical benefits that observed to date.

Endothelial progenitor cells (EPCs) also have therapeutic potential. EPCs can be mobilized from the bone marrow and adventitial tissue surrounding endothelial cells (ECs), and home toward sites of injury. There, they influence the release of vasoactive substances or directly differentiate into mature ECs to regenerate damaged endothelium. Diabetes-related EPC dysfunction is closely linked to the impaired healing response experienced by many patients with diabetic CKD. Circulating EPCs are low in type 2 diabetic patients and the loss of function of these cells may contribute to the vasodegenerative changes observed in diabetic micro- and macrovasculature disease (Schatteman et al., 2000). Therefore, harnessing the vascular reparative properties of EPCs represents a novel treatment for therapeutic revascularization and vascular repair for CKD patients with diabetes.

### Challenges to Reverse Kidney Fibrosis to Promote Repair

A growing number of clinical trials show that MSCs are safe and well tolerated in diabetes (Skyler et al., 2015). The exogenous application of angiogenic-stimulating EPCs has shown promise for treatment of kidney failure, heart disease, and diabetes including retinopathy (Stitt et al., 2013). Both MSCs and EPCs mediate their effects largely through paracrine signaling and therefore require microenvironments that support optimal cell engraftment and proliferation. However, impediments in clinical translation occur due to low cell survival rates following transplantation that limit therapeutic efficacy (Chevalier et al., 2010). In particular, the fibrosis and chronic inflammation hamper cell survival and limit the cell integration into host tissue. Modulation and removal of the fibrotic lesion is therefore crucial to facilitate cell integration. In addition, the low number of transplanted cells retained at the site of injury also hampers stem cell efficacy.

To overcome these limitations, we recently reported a bimodal attack by combining MSC therapy and relaxin (RLX) to combat kidney fibrosis progression and aid in MSC survival (Huuskes et al., 2015). Combined MSCs and RLX administration in an obstructive nephropathy model significantly ameliorated kidney fibrosis, reduced macrophage infiltration, myofibroblast proliferation, and upregulated active MMP-2 compared to either therapy alone. This suggested that rather than inhibiting collagen accumulation, combination therapy induced significant collagen degradation. We provide evidence that RLX may influence MSCs *in vivo* creating a more favorable environment for MSC-mediated repair (Huuskes et al., 2015). Targeting fibrosis resolution and limiting vascular damage may also be beneficial through combination therapy, as kidney function is dependent on adequate organ perfusion.

## Liver Fibrosis

### Epidemiology, Burden of Disease, and Natural History

Globally in 2013, cirrhosis was the 6th cause of life years lost in developed countries; ranging from 5th in Europe and central Asia, to 9th in southeast Asia and Oceania. In the United States, cirrhosis was the 12th leading cause of death overall and the 5th in adults aged 45–54 years (Heron, 2012). Common causes of chronic injury leading to cirrhosis include non-alcoholic steatohepatitis (NASH), alcohol use, and viral hepatitis.

Hepatic fibrosis will progress to cirrhosis in many patients unless the cause of injury is removed. Progressive hepatocyte loss and subsequent disruption of the hepatic vasculature by unregulated ECM expansion result in liver insufficiency characterized by jaundice, coagulopathy, and hypoalbuminemia. Portal hypertension leads to ascites, variceal hemorrhage, and hepatic encephalopathy. The onset of any of these conditions defines hepatic decompensation, which has a significantly higher 1-year mortality than compensated cirrhosis, 20% compared with 5% in one study of 700 patients (Zipprich et al., 2012). In these patients, the only treatment that alters long-term survival is liver transplantation. Unfortunately, not all patients are transplantation candidates and wait-list mortality remains a concern (Toniutto et al., 2016).

### Pathogenesis

Hepatic fibrogenesis involves a dynamic interplay among hepatic stellate cells (HSCs), macrophages, and liver progenitor cells (LPCs). HSCs are pericytes that store vitamin A. During chronic liver injury, they transform to myofibroblasts, acquire a contractile phenotype, and accumulate at sites of injury where they secrete large amounts of ECM including collagen. TGFβ is a major fibrogenic cytokine that triggers HSC activation and ECM production and induces hepatocyte apoptosis (Gressner, 2002). Platelet-derived growth factor (PDGF) is the most potent mitogenic cytokine for HSC (Borkham-Kamphorst et al., 2007). These cytokines are logical targets for drug development. Blocking TGFβ and PDGF signaling has been effective in ameliorating experimental liver fibrosis (Yata et al., 2002; Liu et al., 2011), however, off-target effects hinder clinical development.

Kupffer cells (resident liver macrophages) and recruited circulating monocytes contribute to inflammation, fibrogenesis, and fibrosis resolution. Macrophages are capable of distinct activation states and functions, broadly classified as M1 (classical) or M2 (alternative) (Mantovani et al., 2004). M1 macrophages are classically pro-inflammatory, whereas M2 macrophages are responsible for immunomodulation and wound-healing responses. In addition a fibrolytic macrophage subset (Ly6C^lo^) that produces high levels of matrix metalloproteinases that contribute to ECM degradation has been described (Ramachandran et al., 2012).

LPCs are rare in healthy tissue but proliferate and differentiate into cholangiocytes or hepatocytes during chronic liver injury. The LPC response corresponds with the degree of liver injury (Lowes et al., 1999; Roskams et al., 2003) because, unlike hepatocytes, LPC resist the anti-proliferative actions of TGFβ (Nguyen et al., 2007). LPC express surface markers representative of their primitive, undifferentiated state such as Thy-1 (CD90), prominin (CD133), and pan-cytokeratin. A close physical relationship exists between HSC and LPC suggesting that the two cell types proliferate in tandem as HSC depletion significantly dampens the LPC response (Roskams, 2008; Ruddell et al., 2009). HSC produce soluble factors that increase LPC proliferation and hepatocyte differentiation (Nagai et al., 2002; Lin et al., 2008) and ECM proteins produced by HSC, such as laminin, may activate the LPC response (Kallis et al., 2011). Conversely, LPC produce lymphotoxin (LT), which recruits HSC through paracrine signaling (Ruddell et al., 2009). LPC also recruit macrophages via CCL2 and CX3CL1. Macrophage-derived TNF and LT, in turn, influence LPC response (Viebahn et al., 2010).

### Treatment of Hepatic Fibrosis

The concept that hepatic fibrosis develops from a wound-healing response to chronic injury provides a rational basis for treatment. Diminishing liver injury by inhibiting chronic hepatitis B replication results in significant fibrosis regression in cirrhotic patients (Marcellin et al., 2013). Similar outcomes occur in patients with chronic hepatitis C infection (Hoefs et al., 2011). In diseases without specific therapy, a general anti-fibrotic approach might be useful. However, a recent trial of a monoclonal antibody against lysl-oxidase-like 2, which mediates collagen cross-linkage, was not effective (Meissner et al., 2016). Considering the complex interactions involved in hepatic wound healing, cell-based therapy may provide a strategy to control inflammation, degrade collagen, and promote hepatic parenchymal regeneration. Human clinical trials have utilized MSC with variable cell doses, delivery routes, and administration frequency (**Table 2**). Trial endpoints commonly include liver tests, ascites volume, or clinical scores (Child–Pugh–Turcotte, model for end-stage liver disease). To date, outcomes have yet to translate into clinical practice. Furthermore, there is experimental evidence that bone marrow-derived MSC can contribute to hepatic fibrosis (Russo et al., 2006). MSCs as an anti-fibrotic therapy has been critically reviewed (Haldar et al., 2016).

**Table 2:** Summary of reports from clinical trials assessing safety and efficacy of cell therapies for lung and liver fibrosis.

Study	Number of patient treated/control	Cell type	Route	Number of cells transfused/number of injections	Functional benefit sustained to end of F/U period?	Safety
**Clinical trials in lung fibrosis**
Tzouvelekis et al., 2013	14/0	Autologous adipose stromal cells	Endobronchial	0.5 × 10^6^/kg body weight single injection	No, 12 months	No serious side-effects or complications
Chambers et al., 2014	8/0	Allogeneic placental MSC	Intravenous	1 × 10^6^; 2 × 10^6^ kg body weight single injection	No, 6 months	One chest infection; one IPF exacerbation
Glassberg et al., 2016	9/0	Allogeneic BM MSC	Intravenous	20, 100, or 200 × 10^6^ single injection	Yes, 6 months	No serious side-effects or complications
**Clinical trials in liver fibrosis**
Terai et al., 2006	9/0	Autologous BM	Peripheral IV	2.21–8.05 × 10^9^ Avg. 5.2 × 10^9^	Significant decrease in average CPT at 4 and 24 weeks	All had fever (38°C) at 1 day post-therapy
Couto et al., 2011	8/0	Autologous BM MNC	HA	2–15 × 10^8^ single injection	Yes, 2 months No, 12 months	
Amer et al., 2011	20/20	Hepatocyte lineage from autologous BM MNC	Intrahepatic or intrasplenic	5 mL of cell suspension (2 × 10^6^/mL) single injection	Yes, 6 months	Fever within 24 h after injection in 10 subjects (50%)
Peng et al., 2011	53/105	Autologous BM	HA	10^6^/mL, number transfused not stated	Yes, 3 and 9 months No, 48 months	No serious side-effects or complications
El-Ansary et al., 2012	15/10	BM MNC nine undifferentiated six HC differentiated	Peripheral IV	10^6^/kg (40% HLC, 60% MSC) single injection	Yes, 3 and 6 months	No safety evaluation
Zhang et al., 2012	31/15	Umbilical cord MSC	Peripheral IV	0.5 × 10^6^/kg body weight	Yes, 48 weeks	Four had fever 38°C at 2–6 h
Mohamadnejad et al., 2013	15/12	BM MSC	Peripheral vein (30 min)	195 million (120–295 million) single injection	No difference between treated and control	
Lukashyk et al., 2014	6/0	BM MSC	Intrahepatic	5 mL suspension, 1 × 10^6^/kg single injection	Yes, 1 and 6 months	No safety evaluation
Salama et al., 2014	20/20	G-CSF, autologous BM MSC	Peripheral IV	1 × 10^6^/kg body weight	Yes, 6 months	
Mohamadnejad et al., 2016	18/9	Eight CD133^+^ nine BM MNC	Portal vein	4.7 × 10^6^–9.17 × 10^8^ (averages) two injections	Yes, 3 months No, 6 months	No procedural complications

We studied human amnion epithelial cells (hAECs), fetus-derived stem-like cells that arise prior to gastrulation and are easily isolated from the placenta, which is an abundant and ethically undisputed source. hAEC prevent and reverse inflammation and established fibrosis in immunocompetent animal models of liver injury (Manuelpillai et al., 2010), diminish myofibroblast activation, and skew hepatic macrophages toward a reparative phenotype (Manuelpillai et al., 2012). Similar effects are seen with cell-free conditioned media, suggesting that hAEC release factors responsible for the observed outcomes (Hodge et al., 2014). Liver fibrosis reduction also occurs in hAEC-treated mice given a “Western diet” high in lipids and fructose to model fatty liver disease (unpublished). A phase 1 safety trial is planned in patients with compensated cirrhosis.

## Summary

The global burden of end-stage fibrotic disease can be seen in the impaired survival of patients with IPF, diabetic CKD, and cirrhosis. Fortunately, the pathogenesis of fibrosis in response to injury is relatively well understood and remarkably similar in different organs, suggesting that an integrated approach may be possible. Control or removal of the injury stimulus should be the primary focus in preventing disease progression, yet for many control is incomplete or unachievable, thus the need for a broadly effective anti-fibrotic therapy that targets multiple fibrogenic pathways remains. Cell-based approaches employing stem cells that are easy to isolate and upscale to sufficient quantities for clinical use have been successfully characterized in animal models of organ fibrosis. While the outcomes of early phase clinical trials indicate that cell-based (primarily MSC) therapies are safe, efficacy data remain scarce. Consequently, cell-based therapies remain largely experimental. The lack of robust efficacy data may be due to the heterogeneity of MSC populations as well as limited agreement regarding differentiation state, doses, and administration regimens. Challenges remain in determining the goals of cell therapy – whether to supply sufficient cells to replace damaged parenchyma, to dampen inflammation with the aim of decreasing fibrosis, or to stimulate endogenous progenitor cells and repair processes. Furthermore, the ability to manufacture, transport, and store stem cells in a cost-effective manner must be considered. Clinical trials will continue to inform us about the most effective stem cell types on which to base therapy as well as the optimal dosages necessary to achieve a clinically meaningful reduction in fibrosis-related organ dysfunction.

## Author Contributions

WS contributed the liver fibrosis section; RL contributed the lung fibrosis section; SR contributed the kidney fibrosis section; and all authors reviewed the manuscript and provided critical intellectual input.

## Conflict of Interest Statement

The authors declare that the research was conducted in the absence of any commercial or financial relationships that could be construed as a potential conflict of interest.
